# Asthma improvement in patients treated with dupilumab for severe atopic dermatitis

**DOI:** 10.3389/falgy.2023.1223657

**Published:** 2023-09-11

**Authors:** Marco Dubini, Valentina Benzecry, Federica Rivolta, Andrea Sangalli, Angelo Valerio Marzano, Valerio Pravettoni, Simona Tavecchio, Silvia Mariel Ferrucci

**Affiliations:** ^1^ Department of Internal Medicine, Fondazione IRCCS Ca’ Granda Ospedale Maggiore Policlinico, Milan, Italy; ^2^ Dermatology Unit, Fondazione IRCCS Ca’ Granda Ospedale Maggiore Policlinico, Milan, Italy; ^3^ Department of Pathophysiology and Transplantation, Università degli Studi di Milano, Milan, Italy

**Keywords:** dupilumab, asthma, atopic dermatitis, atopic comorbidities, type 2 disease, allergic asthma, multidisciplinarity

## Abstract

**Introduction:**

Atopic dermatitis (AD) is considered a systemic type 2 immune driven disease, and it is associated to many atopic comorbidities including asthma. The aim of our study was to prospectively evaluate the respiratory outcomes in patients with persistent allergic asthma treated with dupilumab due to severe AD (sAD).

**Methods:**

We enrolled eligible patients with sAD for dupilumab treatment from September 2018 to December 2020. We then selected the subgroup of patients sensitized to perennial allergens. Dupilumab's efficacy and safety on AD and comorbid asthma were assessed at baseline, one month, four months, and then every 4 months up to one year.

**Results:**

A total of 437 patients with sAD were enrolled for dupilumab treatment due to sAD, and 273 reached 48 weeks of therapy. Respiratory outcomes were evaluated in the 85 asthmatic patients with positivity only to perennial allergens. Our patients showed statistically and clinically significant improvement in asthma control (Asthma Control Test and Asthma Control Questionnaire) and airway obstruction parameters (FEV1), in addition to the expected AD-related skin outcomes. Specifically, a significant improvement was achieved at the fourth month of dupilumab therapy, and this trend was maintained up to twelve months, regardless of asthma severity.

**Conclusions:**

Our results showed the overall improvement of the clinical picture that dupilumab offers for patients with severe AD and persistent allergic asthma of any severity, highlighting the importance of a global multidisciplinary approach of type 2 driven disease.

## Introduction

Atopic dermatitis (AD) is associated with impaired skin barrier function and systemic immune dysregulation. It is considered a systemic type 2 immune driven disease with a substantial burden on quality of life, and it is associated to many comorbidities. These are mainly due to the shared pathophysiology and the complex interplay of inflammatory cytokines that have different effects on the skin and other susceptible tissue (1).

AD is often the beginning of the so called “atopic march”, that refers to the propensity for AD to begin early in life, and be followed by the serial occurrence of food allergy, asthma, allergic rhinitis, chronic rhinosinusitis (CRS) with or without nasal polyposis (NP), and eosinophilic esophagitis (2, 3). The main hypothesis for this progression is that, in susceptible individuals, a defect in the skin barrier induces type 2 inflammatory responses to food and inhalant allergens (2). On the other hand, the so-called “inside out” hypothesis, describes the dysregulation of type 2 inflammation as primum movens followed by the skin barrier alteration (4). Epidemiological studies have shown that, not only patients with AD are more susceptible to develop atopic comorbidities, but also their prevalence increases in patients with more severe AD (3). In fact, in adults with AD, the one-year prevalence of self-reported asthma and allergic rhinitis was of 18.7% and 28.45%, respectively (5). Type 2 inflammation pathways are known to contribute to skin and airways barrier disfunction and are driven by type 2 cytokines, as interleukin (IL)-4, IL-5, and IL-13 (6).

During the last decade, a deeper understanding of the pathogenesis of type 2 inflammatory diseases affecting different tissues has resulted in significant therapeutic progress (7, 8). Dupilumab is a fully human monoclonal IgG4 antibody directed against the *α*-subunit of the IL-4 receptor, thereby inhibiting the IL-4 and IL-13 signalling. It has proven to be effective and safe for atopic dermatitis (AD) (9), asthma (10), and other types 2 immunologic signatures (8, 11). In Italy dupilumab was primarily admitted for reimbursement by the Italian Medicines Agency (Agenzia Italiana del Farmaco-AIFA) only for adults (18 years old or older) with severe AD (sAD) (12). In January 2022, dupilumab has also been approved to treat severe asthma and/or NP, and adolescents with severe AD (13).

The aim of our study was to prospectively evaluate the respiratory outcomes in patients with persistent allergic asthma treated with dupilumab due to sAD.

## Materials and methods

Between September 2018 and December 2020 patients aged 18 years and older, with sAD eligible for dupilumab according to the Italian Drug Agency (AIFA) criteria, were enrolled. Inclusion criteria were: sAD with an Eczema Area Severity Index (EASI) score of 24 or greater, and contraindication, inadequate response, or intolerance to cyclosporin A (12). All patients were treated with an initial dose of 600 mg, followed by 300 mg every other week. Patients were assessed for demographics, medical history, comorbid type 2 atopic diseases (i.e., allergic oculorhinitis, asthma, CRS with and without NP, food allergy, and eosinophilic esophagitis), and concomitant medications. In order to study the efficacy of dupilumab on comorbid persistent allergic asthma in these patients, we selected the subgroup of patients sensitized to perennial allergens (dust mites, molds, and animal dander). Dupilumab's efficacy and safety on AD and comorbid asthma were assessed at baseline, one month (T1), four months (T4), and then every 4 months up to one year (T8 and T12, respectively). Throughout the first four months of dupilumab therapy, patients were instructed to maintain their pretreatment therapy for the management of atopic comorbidities. Exclusion criteria were allergen immunotherapy and concomitant therapy with systemic immunosuppressants, other than oral corticosteroid at a daily dose lower than 5 mg of prednisone equivalent.

All procedures were in accordance with the ethical standards of the responsible committee on human experimentation (institutional and national), and with the Helsinki Declaration of 1975, as revised in 2013. Informed written consent was obtained from the patients.

AD was evaluated with the following parameters: EASI score (range: 0–72), Investigator's Global Assessment (IGA) (range: 0–4), Patient-Oriented Eczema Measure (POEM) (range: 0–30), peak score on the Numerical Rating Scale (NRSp) for pruritus (range: 0–10), peak score on the Numerical Rating Scale (NRSs) for sleep (range: 0–10), Dermatology Life Quality Index (DLQI) (range: 0–28) and Hospital Anxiety and Depression Scales (HADS).

The diagnosis and the assessment of severity of allergic asthma were based on a history of variable respiratory symptoms, confirmed by the evidence of variable expiratory airflow limitation and by skin prick test and/or specific IgE positivity to perennial allergens. Asthma was evaluated with pulmonary function testing (Vitalograph, UK), Asthma Control Test (ACT) (range: 0–25), seven-item Asthma Control Questionnaire (ACQ-7) (range: 0–6), and exhaled nitric oxide (FeNO) (NIOX Monitoring System, Aerocrine, Sweden). Asthma exacerbation was defined as requiring either the use of oral corticosteroids for at least 3 days, hospitalization, or emergency room visit (14). Minimal clinically important difference for asthma endpoints was defined on the latest evidence: improvement in ACT and ACQ-7 scores of 3 and 0.5 respectively, and improvement in FEV1 and FeNO of at least 15% and 20% respectively, were considered meaningful (14, 15). Sensitization to inhalant allergens was confirmed with skin prick tests (SPT) (Lofarma, Italy and Stallergenes, France) and/or serum specific IgE (ImmunoCAP System®, Sweden). Peripheral blood eosinophil count and total serum IgE levels were collected at each timepoint.

Patients' characteristics and outcomes were compared using Student's *t*-tests, Wilcoxon tests (in cases of non-normality) for quantitative variables, and Fisher's exact tests for qualitative variables. All statistical analyses were performed with IBM® SPSS® Statistics version 20. The threshold for statistical significance was set at *P* < .05.

## Results

A total of 437 patients with sAD were enrolled for dupilumab treatment and 273 reached 48 weeks of therapy. Out of 273, 153 (56.0%) were male, and the median ± interquartile range (IQR) for age was 38.0 ± 12.0 years. AD was categorized as early-onset in 201 patients (73.6%) and as late-onset in 72 patients (26.4%). Median age at diagnosis was 2.0 ± 11.0 years. At baseline (T0) EASI score was 39.5 ± 12.0, IGA was 4.0 ± 0.0, POEM was 22.0 ± 5.0. Type 2 immune comorbidities were common: allergic oculorhinitis (86.1%), asthma (67.8%), food allergy (26.4%), nasal polyposis (9.5%), eosinophilic esophagitis (0.7%) (Table 1). Almost all patients (96.4%) tested positive for at least one inhalant allergen.

**Table 1:** Demographic and clinical data at the baseline (median ± IQR).

*N* = 273	Median ± IQR
Age (year)	38.0 ± 12.0
Sex, male/female	153 (56.0%)/120 (44.0%)
Age of onset	2.0 ± 11.0
Early onset/late onset	201 (73.6%)/72 (26.4%)
Allergic oculorhinitis	235 (86.1%)
Nasal polyposis	26 (9.5%)
Bronchial asthma	185 (67.8%)
Food allergy	72 (26.4%)
Eosinophilic esophagitis	2 (0.7%)
IgE (KUA/l)	3,600.0 ± 4,200.0
Peripheral eosinophils (cells/mm^3^)	532.0 ± 365.0

Table 2 shows the skin-related outcomes during dupilumab therapy. Initial improvements were already evident after the first month, but they continue to improve up to 12 months follow-up. Of note, at 12 months, the reduction from baseline in the median ± IQR percentage of EASI score was −97.5 ± 23.0 (*P* < .001). All the other parameters also showed a significant reduction: the median ± IQR percentage from baseline in the POEM score was −69.5 ± 8.0 (*P* < .001), in peak score on NRS for pruritus −69.9 ± 12.0 (*P* < .001), in the peak score on NRS for sleep −85.6 ± 12.8 (*P* < .001) and in the DLQI score −81.8 ± 16.2 (*P* < .001). After 12 months of dupilumab therapy, 243 patients (89.0%) achieved ≥75% improvement from baseline as measured by EASI score (EASI-75) (*P* < .001) and 177 (64.8%) achieved ≥90% improvement (EASI-90).

**Table 2:** Ad outcomes over time (median ± IQR).

*N* = 273	T0	T1	T4	T8	T12	*P*-value
EASI score	39.5 ± 12.0	9.0 ± 11.0	4.0 ± 6.0	2.0 ± 4.5	1.0 ± 4.0	<.001
IGA score	4.0 ± 0.0	3.0 ± 1.0	2.0 ± 1.0	1.0 ± 0.0	0.0 ± 0.0	<.001
POEM score	23.0 ± 8.8	9.0 ± 9.0	7.0 ± 8.0	5.0 ± 7.0	5.0 ± 6.0	<.001
NRS pruritus	8.5 ± 3.0	3.0 ± 3.0	3.0 ± 2.0	2.0 ± 4.0	2.0 ± 4.0	<.001
NRS sleep	7.0 ± 3.8	2.0 ± 5.0	0.0 ± 2.0	0.0 ± 1.8	0.0 ± 1.0	<.001
DLQI score	15.5 ± 11.0	5.0 ± 9.0	3.0 ± 5.0	3.0 ± 4.0	2.0 ± 5.0	<.001
HADS-A	8.0 ± 7.8	4.0 ± 6.0	3.0 ± 3.0	3.0 ± 4.5	3.0 ± 4.0	<.001
HADS-D	7.0 ± 5.0	4.0 ± 5.0	3.0 ± 5.0	3.0 ± 4.5	2.0 ± 4.0	<.001

Considering the group of 185 asthmatic patients, 7 patients (3.8%) with negative *in vivo* and vitro tests for inhalant allergens and 94 patients (42.1%) with seasonal allergies were excluded. The effect of dupilumab was evaluated in the remaining group of asthmatic patients (84 patients) with positivity only to perennial allergens.

Table 3 illustrates the respiratory outcomes at each timepoint. Among these 84 patients, 48 (57.1%) were male, the median ± IQR for age was 39.0 ± 15.0 years and for the age of asthma onset was 12.5 ± 11.0 years, the median ± IQR of FEV1 before bronchodilation was 3.08 L ± 1.43 at baseline. Most of the patients (52) were using a low dose of inhaled corticosteroids (61.9%), 36 as maintenance (42.9%) and 16 as-needed (19.0%); 24 patients (28.6%) were using a medium dose of inhaled corticosteroids (9.5%), few patients (8) were using a high total daily dose of inhaled corticosteroids (14). After 1 month (T1) no significant improvement was observed (FEV1 before bronchodilation 3.17 L ± 1.42). Instead, a significant improvement was achieved at the fourth month (T4) (FEV1 before bronchodilation was of 3.35 L ± 1.38) (*P* < .001), and this trend was maintained over time at T8 and T12. Median ± IQR of FEV1 (percent of predicted value before bronchodilation) was 96.3 ± 17.8 at baseline, 97.0 ± 17.8 at T1, 101.8 ± 18.1 at T4 (*P* < .001) and 103.0 ± 20.4 at T12 (*P* < .001). The same trend was observed for ratio FEV1/FVC: at baseline 78.0 ± 14.6, after 1 month 79.9 ± 14.1, after 4 months 82.0 ± 9.7 (*P* < .001), after 12 months 81.8 ± 11.5 (*P* < .001). By studying the flows at the lower lung volumes, we noted a significant improvement already after 1 month of FEF 50% (*P* < .05), and after 4 months for FEF 75% and 25% (*P* < .001).

**Table 3:** Asthma outcomes over time (median ± IQR).

*N* = 84	T0	T1	T4	T8	T12	*P*-value
FEV1 (L)	3.08 ± 1.43	3.17 ± 1.42	3.35 ± 1.38	3.31 ± 1.41	3.36 ± 1.48	<.001
FEV1 (% predicted)	96.3 ± 17.8	97.0 ± 17.8	101.8 ± 18.1	103.2 ± 18.2	103.0 ± 20.4	<.001
FEV1/FVC	78.0 ± 14.6	79.9 ± 14.1	82.0 ± 9.7	82.0 ± 10.9	81.8 ± 11.5	<.001
FEF 75	75.0 ± 33.0	75.3 ± 35.0	91.5 ± 30.0	91.2 ± 30.5	90.2 ± 31.8	<.001
FEF 50	70.0 ± 34.3	70.5 ± 33.8	77.5 ± 33.1	75.5 ± 33.8	77.5 ± 33.8	<.001
FEF 25	70.0 ± 35.8	72.0 ± 41.8	73.8 ± 37.0	75.5 ± 36.9	74.7 ± 34.2	<.001
ACT score	20.0 ± 4.0	22.0 ± 3.0	23.0 ± 2.0	23.5 ± 2.0	24.0 ± 2.0	<.001
ACQ-7 score	1.4 ± 1.1	0.9 ± 0.9	0.6 ± 0.6	0.4 ± 0.6	0.3 ± 0.6	<.001
Controlled asthma	61 (72.2%)	67 (79.8%)	75 (89.3%)	77 (91.7%)	81 (96.4%)	<.001
FeNO (ppb)	35.0 ± 22.3	28.0 ± 16.8	24.0 ± 13.0	22.0 ± 11.5	20.0 ± 11.0	<.001
IgE (KUA/l)c	3,292.0 ± 4,800.0	2,699.0 ± 4,658.0	1,909.0 ± 3,423.0	1,220.0 ± 2,011.3	977.5 ± 2,325.0	<.001
Peripheral eosinophils (cells/mm^3^)	600.0 ± 455.0	750.0 ± 730.0	640.0 ± 550.0	500.0 ± 600.0	490.0 ± 520.0	>.05

Asthma questionnaires also showed a significant improvement after 4 months, and this was maintained over time: the median ± IQR score of ACT significantly improved from 20.0 ± 4.0 at baseline to 23.0 ± 2.0 at T4 (*P* < . 001), and of ACQ-7 showed a reduction from 1.4 ± 1.1 to 0.6 ± 0.6 at T4 (*P* < .001). The improvement of both scores were confirmed at T8 and T12 (*P* < .001).

Likewise, the median ± IQR measure of the exhaled nitric oxide (FeNO) significantly decreased from 35 ± 21 ppb at baseline to 25 ± 12 ppb after 4 months (*P* < .001), 24 ± 10 after 8 months and 22 ± 11 after 12 months.

Over time, the proportion of patients with controlled asthma (ACT ≥ 20) progressively increased: from 72.2% at T0 to 79.8% at T1, 89.3% at T4. Almost all patients (96.4%) showed a controlled asthma at T12. At the same time, the ACQ score gradually decreased, reaching values of less than 0.5 in the majority of patients. Considering the minimal clinically significant difference, the number of patients exceeding this threshold was progressively higher over time: at T12 almost half (48.8%) of patients achieved an improvement of at least 3 points in ACT score, while 65.5% achieved a reduction in ACQ-7 score of at least 0.5.

Furthermore, at baseline a total of 13 patients (15.5%) had developed at least one severe asthma exacerbation during the 12 months prior to dupilumab treatment, instead only 1 patient (1.2%) developed at least one during the treatment period (*P* < .001).

Concurrently with clinical improvement, serum total IgE significantly decreased from baseline over the time. For the median ± IQR of total blood eosinophil count, no significant differences from baseline were found at T12. Even if an initial increment was found after 1 month, the eosinophil count was comparable to baseline at T8.

The overall incidence of adverse events during the 12-month treatment was 41.4% (113/273). The most common adverse effects reported were Dupilumab Facial Redness (DFR) (15.0%), local injection site reactions (13.9%), conjunctivitis (12.1%), and blefaritis (2.9%). Three patients (1.0%) developed *de novo* psoriasis during dupilumab treatment.

Only 4% (12/273) discontinued treatment for different reasons, mainly intolerance to side effects. In one patient dupilumab was stopped as a precaution due to the elevation of the blood eosinophil count up to 18,000/mm^3^ after one month of treatment.

## Discussion

Allergic diseases are dominated by systemic type 2 inflammation with overexpression of cytokines such as IL-4 and IL-13, which, in the skin, regulate the epidermal barrier and the effector phase of the immune response (7, 8). Blocking these two cytokines by dupilumab impacts on the overall AD molecular signature (16). The long-term efficacy in skin-related outcomes of dupilumab is well established. In our study the median reduction of EASI score at 12 months was of −97%, which is in line with other studies (a recent study reported a median EASI percentage score reduction of 84% at 16 weeks and 93% at 52 weeks) (17).

Most of adult patients affected by moderate to severe AD reported one or more comorbid type 2 inflammatory diseases, among which asthma, allergic rhinitis, and food allergy were most common (18). Therefore, the assessment of type 2 comorbidities in AD is of utmost importance.

In asthma, IL-4 plays a main role in regulating type 2 cell expansion and type 2-related cytokine production, as well as, IgE synthesis, while IL-13 has a major role in inducing the clinical features of the disease such as mucus production and airway hyperresponsiveness (11). Type 2 driven comorbidities were highly prevalent in our group of patients with sAD, particularly, the prevalence of asthma yielded nearly 70%, and, among these patients, 30% had uncontrolled asthma. A *post hoc* analysis of more than 2000 patients including LIBERTY AD SOLO 1 (NCT02277743), SOLO 2 (NCT02755649), CHRONOS (NCT02260986), and CAFÉ (NCT02755649), reported that nearly 40% of adult patients with moderate to severe AD also had comorbid asthma; and in this population about 20% were uncontrolled or only partially controlled (ACQ-5 ≥ 0.5) and 15% were uncontrolled (ACQ-5 ≥ 1.0) despite receiving concomitant asthma medications (19).

Our patients showed statistically and clinically significant improvement in asthma control (ACT and ACQ-5 scores) and respiratory parameters, in addition to the expected AD-related skin outcomes. Specifically, a significant improvement was achieved at the fourth month of dupilumab therapy, and this trend was maintained up to twelve months. Of note, only one patient developed one severe exacerbation during the treatment period. The results of our study are consistent with the ACQ improvements observed in AD patients treated with dupilumab at week 16 of the CAFE, SOLO1 and SOLO2, and CHRONOS trials (19). Likewise, another study showed at week 16 a significant improvement of ACT and ACQ-5 scores, a clinically relevant improvement in FEV1 in almost half of the patients, and a reduction of severe exacerbations, nevertheless, FEV1 increase did not yield a statistically significant difference compared to baseline (20).

We also evaluated the improvement in FeNO, which significantly decreased by the fourth month. A study of patients with uncontrolled asthma in dupilumab trials showed a significant correlation between decrease of FeNO and improvement in FEV1 (21). The reduction in IgE serum levels under dupilumab has been interpreted as a marker of IL-4/IL-13 blockade, and type 2 inflammation reduction (22–24).

As reported from previous studies, dupilumab-treated patients had an initial increase in eosinophil count reaching its peak in the first month of therapy, with subsequent slow decrease towards baseline level (25). In our patients, the eosinophil count already diminished by the fourth month and reached near baseline levels by the eighth month. The increase in the blood eosinophil count has been explained by the inhibition of eosinophil migration into tissue because of dupilumab effect on the production of eotaxins and other chemokines. This action results in a transient increase in circulating eosinophil counts (16, 26, 27).

Our study further confirms the safety profile of dupilumab in patients with AD and comorbid asthma, since only a minority of patients experienced mild, non-life-threatening adverse effects: facial redness dermatitis, injection site reactions, and conjunctivitis (28).

Dupilumab treatment confers clinical benefits across multiple type 2 comorbid diseases. In our study improvements were observed in all cutaneous objective and subjective AD scores, and, of relevance, in all respiratory objective and subjective scores in the asthmatic subgroup of patients. This is in accordance with previous results (19, 20), thus highlighting the role of potential monotherapy of these two disorders in selected patients (29). It is worth to underline that the follow-up of the present study was of 12 months. This certainly adds additional evidence to the preexisting literature with an average follow-up of 16 weeks (16, 19, 20, 22). Dupilumab is safe, and its efficacy seems to be prolonged and sustained over time.

The limitations of our study are: the lack of control group, the lack of measurements on other biomarkers, and the lack of evaluation of other atopic comorbidities.

The inhibition of the IL-4/IL-13 axis by dupilumab should be recognized as a multisystemic effect (30, 31). Our results showed the overall improvement of the clinical picture that dupilumab offers for patients with severe AD and persistent allergic asthma of any severity. Thus, we emphasize the importance of a global approach of type 2 driven disease, preferably by a multidisciplinary dedicated team of specialists (32). We believe comorbidities and patient burden should be integrated into the assessment and management of atopic dermatitis, optimizing therapeutic decision-making and improving patient outcomes. Furthermore, this therapeutic opportunity offers the possibility of simplifying the treatment approach by employing a drug that can effectively treat multiple type 2 inflammatory diseases at the same time (33).

## Data Availability

The raw data supporting the conclusions of this article will be made available by the authors, without undue reservation.
